# Association between Glaucoma Progression in Macular Ganglion Cell Complex and Disc Hemorrhage: Differences between Superior and Inferior Hemiretinas

**DOI:** 10.3390/jcm12123996

**Published:** 2023-06-12

**Authors:** Gaku Tachibana, Tomomi Higashide, Koji Nitta, Kazuhisa Sugiyama

**Affiliations:** 1 Department of Ophthalmology, Fukui-Ken Saiseikai Hospital, Fukui 918-8503, Japan; tchbngk@med.kanazawa-u.ac.jp (G.T.); nitta.koji7001@fukui.saiseikai.or.jp (K.N.); 2 Department of Ophthalmology, Kanazawa University Graduate School of Medical Sciences, 13-1 Takara-machi, Kanazawa 920-8641, Japan; ksugi@med.kanazawa-u.ac.jp

**Keywords:** disc hemorrhage, glaucoma progression, ganglion cell complex, visual field, normal-tension glaucoma

## Abstract

Disc hemorrhage (DH) is often associated with glaucoma progression. A vertically asymmetrical pattern is typical of glaucoma progression, but it remains unclear whether the association between DH and glaucoma progression differs between the superior and inferior hemiretinas. We compared the thickness changes of the macular ganglion cell complex (GCC) in the hemiretinas of normal-tension glaucoma patients with or without DH, as well as between hemiretinas positive and negative for DH, during five years. Both the superior and inferior hemiretinas in the DH-positive group had a more negative GCC thickness slope in association with more DH counts compared to those in the DH-negative group. Conversely, only the inferior hemiretina exhibited a significant relationship between GCC thickness slope and DH counts when hemiretinas positive and negative for DH in the DH-positive group were compared. In the superior hemifield, the slope of the total deviation changes in the DH-positive hemifield of the DH-positive group was more negative compared to that of the DH-negative group. The association between DH and glaucoma progression in the macular GCC may be stronger in the inferior hemiretina, suggesting that more attention should be paid to DH in the inferior disc area as a sign of glaucoma progression.

## 1. Introduction

Glaucoma is the most common cause of irreversible blindness worldwide [1]. The global prevalence of glaucoma in individuals aged 40–80 years is expected to increase to more than 100 million by 2040 [2]. Efficient detection and prevention of glaucoma progression are urgently needed to reduce the number of patients with vision loss due to glaucoma. A vertically asymmetric pattern is a characteristic of glaucoma progression. Visual field (VF) defects appear more frequently in the superior hemifield during the early stages of glaucoma [3]. The Glaucoma Hemifield Test, which detects sector-wise differences in the VF sensitivity between the superior and inferior hemifields, is commonly used as a representative criterion for glaucoma diagnosis [4]. Hood et al. proposed the term macular vulnerability zone based on studies using optical coherence tomography (OCT), which emphasizes the presence of areas highly susceptible to glaucomatous structural damage in the inferior macula [5].

A systematic review identified the representative risk factors for glaucoma progression [6]. In addition to higher age being a definite risk factor for progression in patients with open-angle glaucoma, the presence of disc hemorrhage (DH) was definitely associated with rapid progression, especially in patients with normal-tension glaucoma (NTG). DH often occurs in association with the structural and/or functional progression of glaucoma. Landmark glaucoma studies, such as the Collaborative Normal Tension Glaucoma Study and Early Manifest Glaucoma Trial, have identified DH as a significant risk factor for VF progression in patients with glaucoma [7,8]. Regarding structural progression, photographic enlargement of retinal nerve fiber layer (RNFL) defects is closely associated with DH [9]. Recently, studies using OCT reported faster thinning of the circumpapillary RNFL, neuroretinal rim, macular retinal ganglion cell (RGC), plus the inner plexiform layer (GCIPL), a representative thickness parameter of the macular inner retinal layers, in association with the occurrence of DH at the site of structural changes [10,11,12,13,14,15,16,17].

Although the exact pathogenesis of DH is still elusive, the association between DH occurrence and factors related to vascular or hemodynamic impairment such as choroidal microvascular dropout [18], migraine [19,20], high [19,21] or low [20] blood pressure, and nail bed hemorrhage [22] supports the vascular hypothesis of DH origin. Conversely, the intimate relationship between DH location and focal structural changes such as RNFL defects [9,23] and focal lamina cribrosa defects [24] indicates that DH may result from focal mechanical damages to microvessels.

DH is found heterogeneously around the optic disc, most commonly in the inferotemporal rim area, which is a vulnerable disc region [25,26]. The concordance of the most frequent disc area between DH and structural progression is reasonable, given that DH indicates the site of faster glaucoma progression. Conversely, the superotemporal disc area was the second most frequent site of DH. Several studies have highlighted the differences in the patterns of RNFL defects between the superior and inferior hemiretinas. Choi et al. reported that superior RNFL defects associated with inferior VF loss were wider and closer to the horizontal meridian of the optic disc than inferior defects associated with superior VF loss, particularly in patients with central VF loss [27]. Hood et al. examined OCT scans of the circumpapillary RNFL and macular GCIPL in patients with early glaucoma and showed that the RNFL defect pattern was often shallow and widespread in eyes with inferior VF loss, whereas it was typically deep and localized in eyes with superior VF loss [28]. Accordingly, the relationship between DH and structural glaucoma progression may differ between the superior and inferior hemiretinas.

Higashide et al. reported that photographic structural progression, which was mostly detected as widening of RNFL defects, occurred comparably in both the superior and inferior hemiretinas in a 3-year prospective study [29]. Nonetheless, the progression sites in the superior hemiretina were less frequently associated with DH than those in the inferior hemiretina. However, no studies using OCT have addressed whether the rate of structural progression is comparably accelerated between the superior and inferior hemiretinas at the site of DH occurrence. If rapid progression at the DH site is specific to the inferior hemiretina, the mechanism of structural progression may differ depending on the disc area. Furthermore, attention should be paid to DH as a sign of accelerated progression especially for DHs in the inferior disc area.

To address this issue, we compared the rate of thickness changes in the macular ganglion cell complex (GCC) per hemiretina in patients with NTG during a study period of five years between the no-DH and DH groups, and between the DH (+) and DH (−) hemiretinas in the DH group. This study focused on NTG, given that DH is more frequently found in patients with NTG than in those with other types of glaucoma [26] and the presence of DH is a definite risk factor for glaucoma progression in patients with NTG [6].

## 2. Materials and Methods

The study was conducted in accordance with the tenets of the Declaration of Helsinki and approved by the Institutional Review Board of Fukui-ken Saiseikai Hospital. Patient consent was obtained through an opt-out method instead of a written form from each participant, given the retrospective nature of the study protocol.

We retrospectively reviewed the charts of patients with glaucoma who were diagnosed and/or treated at the Glaucoma Service of the Department of Ophthalmology in Fukui-ken Saiseikai Hospital between 1997 and 2011. The study period for evaluating glaucoma progression using OCT data was between January 2012 and October 2016. We compared glaucoma progression in patients with NTG between the no-DH and DH groups. Patients in the no-DH group were defined as those who had no DH in either eye not only during the study period but also prior to the study period, which was determined according to the medical records and evaluation of fundus photographs. Patients in the DH group were defined as those with DH in either eye during at least one visit during the study period.

The eligibility criteria for the study participants were as follows: patients diagnosed with NTG who had regular follow-up visits every three months, best-corrected visual acuity ≥ 0.5, mean deviation ≥ −12 dB in the visual field (VF) test, and axial length ≤ 26.5 mm at the baseline visit, that is, the first visit in 2012. NTG was diagnosed according to the baseline data if both eyes had an untreated intraocular pressure (IOP) of ≤21 mmHg as measured by Goldmann applanation tonometry at >3 visits; gonioscopically normal open angle; at least one eye had glaucomatous fundus abnormalities, i.e., enlargement of optic disc cupping, thinning of neuroretinal rim, or RNFL defects; and reproducible VF abnormalities corresponding to the structural changes. The VF was considered to be abnormal when one of the following conditions were present: a cluster of three or more points with *p* < 5% and at least one point with *p* < 1% in the pattern deviation probability plot, a pattern standard deviation of less than 5%, or a glaucoma hemifield test result outside normal limits. The exclusion criteria were ocular findings suggestive of secondary glaucoma, other ocular or systemic diseases that may affect the VF or OCT data, and incisional glaucoma surgeries before or during the study period. If both eyes were eligible, one eye was randomly chosen.

**Figure 1:**
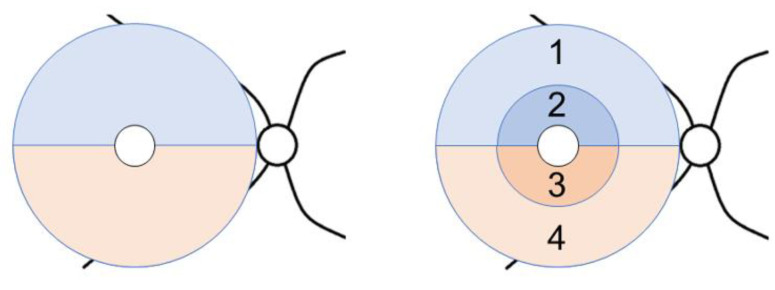
Analytical areas of ganglion cell complex thickness in the macula. Superior (**blue**) and inferior (**pink**) hemiretinas. Superior outer (**1**), superior inner (**2**), inferior inner (**3**), and inferior outer (**4**) sectors.

VFs were examined every six months using the Humphrey Field Analyzer 24-2 Swedish Interactive Thresholding Algorithm program (Carl Zeiss Meditec, Dublin, CA, USA). Only reliable test results with a fixation loss of <20%, false-positive rates of <33%, and false-negative rates of <33% were studied. The total deviation (TD, dB) in the superior and inferior hemifields was evaluated. OCT imaging of the macula was performed every six months using an RS3000 Advance SD-OCT (NIDEK, Gamagori, Japan), which consisted of 128 vertical B-scans, each of which had 512 A-scans over a 9 × 9 mm square area centered on the fovea. Only high-quality images with a signal strength index ≥6, no segmentation errors, and no motion artifacts were studied. GCC thickness was measured in a 9 mm diameter circle area centered on the fovea. The area was divided into superior and inferior hemiretinal areas or into four sectors that consisted of the superior outer, superior inner, inferior outer, and inferior inner areas. The inner and outer areas were the default setting of the OCT device, and were defined using three concentric circles with diameters of 1.5, 4.5, and 9 mm, respectively (Figure 1). The inner area roughly corresponds to an elliptical annulus for GCIPL thickness measurement in the Cirrus HD-OCT (inner vertical and horizontal axes of 1.0 mm and 1.2 mm, respectively; outer vertical and horizontal axes of 4.0 mm and 4.8 mm, respectively; Carl Zeiss Meditec, Dublin, CA, USA) [30].

At each visit every three months, the optic disc was observed through a 14-diopter lens without mydriasis, and fundus photographs with pupil dilation were taken when DH was suspected. Regardless of the results of the fundus examination, photographs of the optic disc were taken at least every six months using a stereo fundus camera (nonmyd WX, Kowa Company, Ltd., Nagoya, Japan). DHs were defined as splinter- or flame-shaped hemorrhages located in association with the optic disc margin, which were irrelevant to other pathologies such as optic disc edema, papillitis, diabetic retinopathy, or retinal vein occlusion. The DH location was sorted into the superior and inferior halves of the optic disc. The hemiretina containing the DH (+ or −) disc location was designated as the DH (+ or −) hemiretina, and the hemifield corresponding to the DH (+ or −) hemiretina was designated as the DH (+ or −) hemifield.

### Statistical Analysis

Numerical variables were compared between the no-DH and DH groups using Student’s t-test. However, a mixed effects model was used when the data were nested. For example, for the variable of “both hemiretinas combined” which contained both superior and inferior hemiretinas, a mixed-effects model was appropriate to account for the correlation between the superior and inferior hemiretinas in the same eye. The thickness changes of the GCC (i.e., thickness slope) were determined using mixed-effects models accounting for potential confounders (i.e., baseline GCC thickness and signal strength index), repeated measurements, and correlation within the same eye. The coefficient of the OCT measurement time from the beginning of the study period (years) indicates the slope of the change in thickness (μm/year). TD changes (i.e., TD slope) were determined using mixed-effects models that accounted for baseline TD values, repeated measurements, and correlations within the same eye. The coefficient of the VF measurement time from the beginning of the study period (years) indicated the slope of the TD change (dB/year). The number of visits with DH was used as a numerical variable to evaluate whether the frequency of DH appearance had any effect on glaucoma progression. When the interaction term DH counts × time (i.e., the time from the beginning of the study period) was significantly negative, more DH appearances were associated with more rapid progression. To control for family-wise error rates in multiple testing, only *p*-values that were significant using the Benjamini-Hochberg method with a false discovery rate of 5% were deemed statistically significant.

## 3. Results

### 3.1. Patient Characteristics

Among 379 patients with NTG whose medical charts were examined, 60 eyes of 60 patients were enrolled in this study: 28 eyes in the no-DH group and 32 eyes in the DH group. At baseline of the study period, mean age was 68.0 ± 9.3 years. IOP before glaucoma treatment and mean IOP during the study period were 14.6 ± 2.6 and 11.7 ± 1.7 mmHg, respectively. Mean medication score was 1.0 ± 0.8 at the beginning of the study period, which remained almost unchanged until the end of the study period. As shown in Table 1, most demographic variables of the study patients, including the mean and standard deviation of IOP during the study period and medication scores, were not significantly different between the no-DH and DH groups. However, the number of eligible OCT measurements was significantly higher in the DH group than in the no-DH group (*p* = 0.002). In the DH group, DH was detected in the superior or inferior halves of the optic disc in 13 and 22 eyes, respectively. DH was detected in both hemiretinas of three eyes. The DHs were more frequently detected in the inferior half than in the superior half of the optic disc (*p* = 0.047).

### 3.2. GCC Thickness Changes

**Figure 2:**
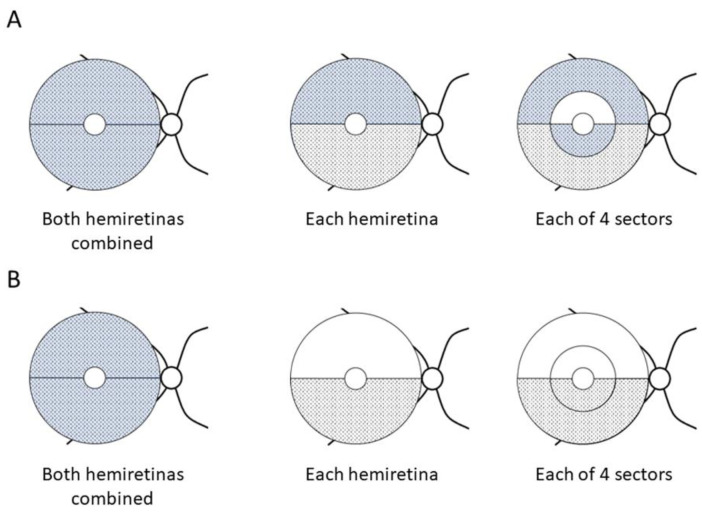
Comparisons of thickness changes in the ganglion cell complex. (**A**) Comparison between the DH and no-DH groups. (**B**) Comparison between the DH (+) and DH (−) hemiretinas in the DH group. The blue area had a significant association between the presence of DH and the more negative thickness slope. Conversely, the patterned area had a significant association between more DH counts and the more negative thickness slope. DH = disc hemorrhage.

At baseline, the GCC thickness in the no-DH group was not significantly different between the superior and inferior hemiretinas (Table S1). Conversely, the GCC in the inferior hemiretina was significantly thinner than that in the superior hemiretina in the DH group (*p* < 0.001). The difference was more significant in DH (−) hemiretinas (*p* < 0.001) than in DH (+) hemiretinas (*p* = 0.053). In the DH group, the GCC thickness in DH (+) hemiretinas was not significantly different from that in DH (−) hemiretinas. When comparing the no-DH and DH groups, the GCC thickness was not significantly different between the two groups in both hemiretinas combined, superior or inferior hemiretinas, DH (−) hemiretinas, or any of the four sectors. Comparisons of the GCC thickness changes between the no-DH and DH groups are shown in Table 2 and Figure 2A. The slope of the GCC thickness changes in both hemiretinas combined was significantly more negative in the DH (+) hemiretinas of the DH group than in those of the no-DH group (*p* = 0.007). Similarly, the GCC thickness slopes in the superior hemiretina, superior outer sector, and inferior inner sector were significantly more negative in the DH (+) hemiretina of the DH group than in the no-DH group (*p* = 0.014, 0.018, and 0.001, respectively). More DH counts during the study period were significantly associated with more rapid progression in both hemiretinas combined (*p* < 0.001), superior (*p* = 0.017), inferior hemiretinas (*p* = 0.001), superior outer (*p* = 0.021), inferior outer (*p* = 0.041), and inferior inner sectors (*p* < 0.001) than in the no-DH group. Comparisons of the GCC thickness changes between the hemiretinas with and without DH in the DH group are shown in Table 3 and Figure 2B. The GCC thickness slope in both hemiretinas combined was more negative in DH (+) hemiretinas than in DH (−) hemiretinas (*p* = 0.004). Higher DH counts during the study period were significantly associated with more rapid progression not only in both hemiretinas combined (*p* < 0.001) but also in the inferior hemiretinas (*p* < 0.001), inferior outer (*p* < 0.001), and inferior inner sectors (*p* = 0.001).

### 3.3. TD Changes

At baseline, the TD in both hemifields combined, superior or inferior hemifield, DH (−) hemifield, DH (−) superior or inferior hemifield was not significantly different between the no-DH and DH groups (Table S2). Comparisons of the TD changes between the no-DH and DH groups are shown in Table 4. The slope of the TD changes in both hemifields combined was significantly more negative in the DH (+) hemifield of the DH group than in the hemifield of no-DH group (*p* = 0.005). Similarly, the TD slope in the superior hemifield was significantly more negative in the DH (+) hemifield of the DH group than in the no-DH group (*p* = 0.008). DH counts during the study period were not significantly associated with the rate of TD progression in either the combined hemifield or the superior or inferior hemifield. Comparisons of the TD slopes between the hemifields with and without DH in the DH group are presented in Table 5. The TD slope was not significantly different between the DH (+) and DH (−) hemifields in combined hemifield, the superior or inferior hemifield. DH counts during the study period were not significantly associated with the rate of TD progression in either the combined hemifield or the superior or inferior hemifield.

## 4. Discussion

In the current study, we studied the changes in GCC thickness over five years per hemiretina to compare the differences between the superior and inferior hemiretinas in patients with NTG. The association between DH and GCC thinning was compared between the DH (+) hemiretinas (DH group) and the corresponding hemiretinas in the eyes of patients without DH (no-DH group), or between the DH (+) and DH (−) hemiretinas in eyes with DH (DH group). The influence of DH was evaluated using two variables: (1) a categorical variable of the presence or absence of DH and (2) a numerical variable of the number of visits with DH. Many studies have examined the significance of DH recurrence in the progression of glaucoma and have reported conflicting results [31,32,33,34,35,36,37,38]. Given that most DHs last at least 4 weeks and only one-third of them persist for 12 weeks or more [39], even studies with frequent follow-up visits of 3 months may miss approximately one-half of the DHs [38]. Therefore, cases with a single DH in most previous studies may have included a considerable proportion of cases with recurrent DH. Instead, the number of visits with DH should not be seriously affected by the underestimation of the true DH occurrence if the follow-up visits are regular and uniform for all study participants. Using this variable, Higashide et al. reported that more visits with DH at the progressive disc location were significantly associated with a shorter time to progression from baseline in a 3-year prospective study with 3-monthly follow-up visits [29].

We showed that the GCC thickness slope per hemiretina was significantly more negative in association with the presence of DH or with more DH appearance when comparing the DH (+) hemiretinas of the DH group and the hemiretinas of the no-DH group. These results agree with those of studies that examined thickness changes in the GCIPL [16,17]. An association between DH counts and GCC thinning was observed in both the superior and inferior hemiretinas when comparing the DH and no-DH groups, but this finding was observed only in the inferior hemiretina when comparing DH (+) and DH (−) hemiretinas in the DH group. These results indicate that the association between DH appearance and ongoing glaucoma progression in macular GCC may be closer in the inferior hemiretina than in the superior hemiretina. One possible explanation for this finding is the structural difference in the GON between the superior and inferior hemiretinas. Hood et al. reported that the pattern of RNFL defects is often shallow and widespread in the superior hemiretina, whereas it is typically deep and localized in the inferior hemiretina [28]. Given that DH often occurs on the border of RNFL defects [23], a more abrupt structural difference across the defect border in the inferior hemiretina is more likely to have a DH appearance than that in the superior hemiretina, as shown in this and previous studies [25,26,29]. A higher number of DH occurrences in the inferior hemiretina would result in a stronger correlation with GCC changes. Another possibility is that the pathogenesis of GON may differ between the two hemiretinas. Previous reports have demonstrated that inferior VF loss, compared with superior RNFL defects, is more frequently observed in glaucoma patients with diabetes or ischemic changes in the brain, suggesting that the superior hemiretina is more susceptible to ischemic changes [40,41]. Higashide et al. reported that structural progression independent of DH was more common in the superior hemiretina than in the inferior hemiretina [29]. Thus, the more frequent structural progression in the absence of DH may have resulted in a weaker association between DH and GCC thinning in the superior hemiretina.

We further analyzed the GCC thickness changes in the four sectors. In comparisons between the no-DH and DH groups, GCC thinning in the superior outer sector and the inferior inner sector was significantly associated with both the presence of DH and increased DH appearance. These results are reasonable, given the differences in the trajectories of the retinal nerve fiber bundles between the superior and inferior hemiretinas. Jansonius et al. reported that more clock hours of the superior half of the optic disc than of the inferior half were devoted to the fovea in a mathematical model describing retinal nerve fiber bundle trajectories [42]. Accordingly, the superior arcuate nerve fibers entering the 11 o’clock disc location, the second most frequent site of DHs, ran more peripherally than the inferior fibers belonging to the vertically line-symmetric disc location (i.e., 7 o’clock, the most common site of DH occurrence) [29,42]. Given the superior outer sector locates outside of the “Cirrus” GCIPL analytical area, the difference in superior macular structural changes between no-DH and DH groups might not have been detected using GCIPL thickness slope instead of GCC thickness slope. Similarly, the diagnostic ability of peripheral macular thickness for early glaucoma was significantly worse when using GCIPL thickness than when using GCC thickness [43]. The diagnostic ability of the GCIPL thickness for glaucoma was significantly inferior to that of the peripapillary RNFL thickness in eyes with inferior hemifield defects (i.e., superior macular damage), whereas both parameters were equivalent for superior hemifield defects (i.e., inferior macular damage) [44].

In addition to evaluating vertical asymmetry in macular structural changes, we also studied functional changes using the TD slope of each hemifield. The presence of DH was significantly associated with a more negative TD slope in the DH (+) hemifield in the DH group than in the hemifield of the no-DH group. The DH counts during the study period were not associated with a worse TD slope of the affected hemifield in any comparison, corroborating previous reports [31,33,34,35,36]. A significant association between DH and TD deterioration was only observed in the superior hemifield (i.e., the inferior hemiretina), which is in agreement with the predominance of the inferior hemiretina over the superior hemiretina in the relationship between DH and GCC thinning.

This study has several limitations. Bias due to the retrospective study design and small sample size may have affected the results. Data collection from a single site may have limited the generalizability of the results. These results may not be applicable to primary open-angle glaucoma with a higher IOP or to patients of other ethnicities or races. The enhancement of glaucoma medication after the occurrence of DH may decrease glaucoma progression [14]. However, the IOP level and medication scores during the study period did not differ between the no-DH and DH groups. The no-DH group might have included patients with undetected DHs, considering the short duration of DH [39]. However, the interval of three months for DH observation in this study is comparable to that adopted in other studies on DHs [11,14,15,16,17,18,20,22,24,29,32,33,34,36,37,38]. Although we did not examine the association between DH and systemic diseases or drug usage, systemic hypertension [19,21], diabetes [19,45] and use of aspirin [45] were reported to be significantly associated with DH occurrence.

In conclusion, DH appearance and accelerated progression at the DH site, as assessed by macular GCC thickness, were more strongly associated in the inferior hemiretina than in the superior hemiretina in patients with NTG. These results indicate that the mechanism of structural progression may differ depending on the disc area. More attention should be paid to DH in the inferior disc area as a sign of glaucoma progression.

## Figures and Tables

**Table 1:** Patient characteristics in each group.

	No DH (n = 28)	DH (n = 32)	*p*-Value, between Groups
Age (year)	67.9 ± 8.1	68.1 ± 10.3	0.92
Gender (Male/Female)	16/12	17/15	0.76 *
Right/left eye	16/12	16/16	0.58 *
Number of visits with DH in superior/inferior half of the disc ^†^	0/0	0.7 ± 1.3/1.5 ± 1.8	NA
*p*-Value, between hemiretinas	NA	0.047 ^‡^	
Spherical equivalent (D)	−2.8 ± 2.8	−1.9 ± 3.0	0.25
Axial length (mm)	24.6 ± 1.2	24.1 ± 1.1	0.11
IOP before treatment (mmHg)	14.7 ± 2.8	14.5 ± 2.5	0.80
IOP at the beginning of the study period (mmHg)	12.1 ± 2.4	12.4 ± 1.8	0.51
Mean IOP ^§^ (mmHg)	11.7 ± 1.9	11.7 ± 1.5	0.85
Mean IOP reduction ^§^ (%)	18.6 ± 13.9	18.3 ± 11.0	0.91
SD of IOP ^§^ (mmHg)	1.35 ± 0.35	1.35 ± 0.30	0.93
Medication score at the beginning of the study period	0.82 ± 0.77	1.16 ± 0.88	0.13 ^†^
Medication score at the end of the study period	0.82 ± 0.67	1.03 ± 0.93	0.49 ^†^
Baseline MD (dB)	−3.6 ± 4.1	−3.5 ± 3.4	0.82 ^†^
Number of OCT measurements ^†^	5.4 ± 1.1	6.9 ± 2.2	0.002 ^† ||^
Number of VF measurements ^†^	7.7 ± 1.2	9.0 ± 3.0	0.13 ^†^

DH = disc hemorrhage, NA = not applicable, IOP = intraocular pressure, SD = standard deviation, MD = mean deviation, and VF = visual field. * Chi-square test. ^†^ Wilcoxon rank-sum test. ^‡^ Mixed-effects models accounting for the correlation between two hemiretinas within the same eye. Other *p*-values were by Student’s *t*-test. ^§^ Based on IOP values measured during the study period. ^||^ Significant using the Benjamini-Hochberg method for controlling the false discovery rate at 5%.

**Table 2:** Comparisons of thickness changes * in ganglion cell complex (μm/year) between groups.

	DH	No DH (n = 28) Coef *, *p*-Value	DH (n = 32) Coef *, *p*-Value	^†^*p*-Value, between Groups	^‡^*p*-Value, Effect of Number of Visits with DH, between Groups
Both hemi-retinas combined	+		−1.04 ± 0.19, *p* < 0.001 (n = 35)	0.007 ^§^	<0.001 ^§^
	−	−0.48 ± 0.11, *p* < 0.001 (n = 56)	−0.36 ± 0.16, *p* = 0.028 (n = 29)	0.400	NA
Superior hemiretina	+		−1.11 ± 0.32, *p* = 0.001 (n = 13)	0.014 ^§^	0.017 ^§^
	−	−0.51 ± 0.13, *p* < 0.001 (n = 28)	−0.52 ± 0.22, *p* = 0.016 (n = 19)	0.951	NA
Inferior hemiretina	+		−0.95 ± 0.23, *p* < 0.001 (n = 22)	0.097	0.001 ^§^
	−	−0.45 ± 0.18, *p* = 0.013 (n = 28)	−0.02 ± 0.18, *p* = 0.915 (n = 10)	0.143	NA
Superior outer sector	+		−1.26 ± 0.36, *p* = 0.001 (n = 13)	0.018 ^§^	0.021 ^§^
	−	−0.62 ± 0.14, *p* < 0.001 (n = 28)	−0.65 ± 0.25, *p* = 0.009 (n = 19)	0.919	NA
Superior inner sector	+		−1.16 ± 0.32, *p* < 0.001 (n = 13)	0.051	0.125
	−	−0.64 ± 0.14, *p* < 0.001 (n = 28)	−0.54 ± 0.18, *p* = 0.003 (n = 19)	0.615	NA
Inferior outer sector	+		−0.67 ± 0.22, *p* = 0.003 (n = 22)	0.668	0.041 ^§^
	−	−0.51 ± 0.22, *p* = 0.018 (n = 28)	0.10 ± 0.19, *p* = 0.616 (n = 10)	0.073	NA
Inferior inner sector	+		−1.76 ± 0.37, *p* < 0.001 (n = 22)	0.001 ^§^	<0.001 ^§^
	−	−0.36 ± 0.15, *p* = 0.019 (n = 28)	0.31 ± 0.29, *p* = 0.291 (n = 10)	0.650	NA

* Coefficients for the OCT measurement time from the beginning of the study period (years) in the mixed-effects models accounting for baseline GCC thickness, signal strength index, repeated measurements, and correlation within the same eye. Significance of the coefficients are indicated by *p*-values. ^†^ Significance of the interaction term between OCT measurement time and a categorical variable of groups. ^‡^ Significance of the interaction term between OCT measurement time and a numerical variable of number of visits with DH in the corresponding hemiretina during the study period. DH = disc hemorrhage; NA = not applicable. ^§^ *p*-Values significant using the Benjamini-Hochberg method for controlling the false discovery rate at 5%.

**Table 3:** Comparisons of thickness changes in ganglion cell complex (μm/year) between hemiretinas with and without DH (DH group, n = 32).

	DH	Coef *, *p*-Value	^†^*p*-Value, DH+ vs. DH− Hemi-Retinas	^‡^*p*-Value, Effect of Number of Visits with DH
Both hemiretinas combined	+ (n = 35)	−1.04 ± 0.19, *p* < 0.001	0.004 ^§^	<0.001 ^§^
	− (n = 29)	−0.36 ± 0.16, *p* = 0.028		
Superior hemiretina	+ (n = 13)	−1.11 ± 0.32, *p* = 0.001	0.035	0.063
	− (n = 19)	−0.52 ± 0.22, *p* = 0.016		
Inferior hemiretina	+ (n = 22)	−0.95 ± 0.23, *p* < 0.001	0.015	<0.001 ^§^
	− (n = 10)	−0.02 ± 0.18, *p* = 0.915		
*p*-value, between hemiretinas (DH+)		0.383		
*p*-value, between hemiretinas (DH−)		0.133		
Superior outer sector	+ (n = 13)	−1.26 ± 0.36, *p* = 0.001	0.075	0.088
	− (n = 19)	−0.65 ± 0.25, *p* = 0.009		
Superior inner sector	+ (n = 13)	−1.16 ± 0.32, *p* < 0.001	0.050	0.137
	− (n = 19)	−0.54 ± 0.18, *p* = 0.003		
Inferior outer sector	+ (n = 22)	−0.67 ± 0.22, *p* = 0.003	0.032	<0.001 ^§^
	− (n = 10)	0.10 ± 0.19, *p* = 0.616		
Inferior inner sector	+ (n = 22)	−1.76 ± 0.37, *p* < 0.001	0.030	0.001 ^§^
	− (n = 10)	−0.31 ± 0.29, *p* = 0.291		

* Coefficients for the OCT measurement time from the beginning of the study period (years) in the mixed-effects models accounting for baseline GCC thickness, signal strength index, repeated measurements, and correlation within the same eye. Significance of the coefficients are indicated by *p*-values. ^†^ Significance of the interaction term between OCT measurement time and a categorical variable of whether DH was present or not in the corresponding hemiretina. ^‡^ Significance of the interaction term between OCT measurement time and a numerical variable of number of visits with DH in the corresponding hemiretina during the study period. DH = disc hemorrhage; ^§^ *p*-Values significant using the Benjamini-Hochberg method for controlling the false discovery rate at 5%.

**Table 4:** Comparisons of total deviation changes * (dB/year) between groups.

	DH	No DH (n = 28) Coef *, *p*-Value	DH (n = 32) Coef *, *p*-Value	^†^*p*-Value, between Groups	^‡^*p*-Value, Effect of Number of Visits with DH, between Groups
Both hemifieldscombined	+		−0.44 ± 0.11, *p* < 0.001 (n = 35)	0.005 ^§^	0.138
	−	−0.11 ± 0.06, *p* = 0.06 (n = 56)	−0.27 ± 0.07, *p* < 0.001 (n = 29)	0.112	NA
Superior hemifield	+		−0.51 ± 0.16, *p* = 0.001 (n = 22)	0.008 ^§^	0.052
	−	−0.07 ± 0.08, *p* = 0.038 (n = 28)	−0.20 ± 0.18, *p* = 0.258 (n = 10)	0.527	NA
Inferior hemifield	+		−0.30 ± 0.14, *p* = 0.031 (n = 13)	0.318	0.336
	-	−0.16 ± 0.09, *p* = 0.061 (n = 28)	−0.29 ± 0.07, *p* < 0.001 (n = 19)	0.251	NA

* Coefficients for the visual-field measurement time from the beginning of the study period (years) in the mixed-effects models accounting for baseline total deviation values, repeated measurements, and correlation within the same eye. Significance of the coefficients are indicated by *p*-values. ^†^ Significance of the interaction term between visual-field measurement time and a categorical variable of groups. ^‡^ Significance of the interaction term between visual-field measurement time and a numerical variable of number of visits with DH in the corresponding hemiretina during the study period. DH = disc hemorrhage, NA = not applicable. ^§^ *p*-Values significant using the Benjamini-Hochberg method for controlling the false discovery rate at 5%.

**Table 5:** Comparisons of total deviation changes (dB/year) between hemifields with and without DH (DH group).

	DH	Coef *, *p*-Value	^†^*p*-Value, DH+ vs. DH− Hemi-Fields	^‡^*p*-Value, Effect of Number of Visits with DH
Both hemifields combined	+ (n = 35)	−0.44 ± 0.11, *p* < 0.001	0.244	0.765
	− (n = 29)	−0.27 ± 0.07, *p* < 0.007		
Superior hemifield	+ (n = 22)	−0.51 ± 0.16, *p* = 0.001	0.321	0.494
	− (n = 10)	−0.20 ± 0.18, *p* = 0.258		
Inferior hemifield	+ (n = 13)	−0.30 ± 0.14, *p* = 0.031	0.871	0.098
	− (n = 19)	−0.29 ± 0.07, *p* < 0.001		
*p*-value, between hemifields (DH+)		0.392		
*p*-value, between hemifields (DH−)		0.577		

* Coefficients for the visual-field measurement time from the beginning of the study period (years) in the mixed-effects models accounting for baseline total deviation values, repeated measurements, and correlation within the same eye. Significance of the coefficients are indicated by *p*-values. ^†^ Significance of the interaction term between visual-field measurement time and a categorical variable of whether DH was present or not in the corresponding hemiretina. ^‡^ Significance of the interaction term between visual-field measurement time and a numerical variable of number of visits with DH in the corresponding hemiretina during the study period. DH = disc hemorrhage. None of the *p*-values were significant using the Benjamini-Hochberg method for controlling the false discovery rate at 5%.

## Data Availability

The data supporting the findings of this study are available from the corresponding author upon request.

## Supplementary Materials

The following supporting information can be downloaded at: https://www.mdpi.com/article/10.3390/jcm12123996/s1, Table S1: Baseline thickness of ganglion cell complex (µm); Table S2: Baseline total deviation (dB).
