# Supplementary material for: Association between Glaucoma Progression in Macular Ganglion Cell Complex and Disc Hemorrhage: Differences between Superior and Inferior Hemiretinas
Source: J Clin Med. 2023 Jun 12;12(12):3996. doi: 10.3390/jcm12123996 (PMC10299411; doi:10.3390/jcm12123996)
Supplement: Supplementary file 1 [file jcm-12-03996-s001.zip › jcm-2404991-supplementary.pdf]

**Table S1**. Baseline thickness of ganglion cell complex (μm)

| | no DH (n=28) | DH (n=32) | p-value, between groups |
|---|---|---|---|
| Both hemiretinas combined | 75.5±13.0 (n=56) | 76.2±12.0 (n=64) | 0.75* |
| Superior hemiretina | 77.6±14.0 (n=28) | 81.7±10.4 (n=32) | 0.19 |
| Inferior hemiretina | 73.4±11.9 (n=28) | 70.8±11.0 (n=32) | 0.37 |
| p-value, between hemiretinas | 0.224* | <0.001*§ | |
| DH (+) hemiretina | NA | 74.6±10.9 (n=35) | NA |
| DH (-) hemiretina | 75.5±13.0 (n=56) | 78.2±13.1 (n=29) | 0.35* |
| p-value, between hemiretinas | NA | 0.196* | |
| DH (+) superior hemiretina | NA | 78.9±9.1 (n=13) | NA |
| DH (+) inferior hemiretina | NA | 72.0±11.3 (n=22) | NA |
| DH (-) superior hemiretina | 77.6±14.0 (n=28) | 83.6±11.0 (n=19) | 0.12 |
| DH (-) inferior hemiretina | 73.4±11.9 (n=28) | 68.0±10.6 (n=10) | 0.21 |
| p-value, between superior and inferior hemiretinas (DH+) | NA | 0.053 | |
| p-value, between superior and inferior hemiretinas (DH-) | 0.224* | P<0.001§ | |
| p-value, between DH+ and DH- hemiretinas (superior) | NA | 0.212 | |
| p-value, between DH+ and DH- hemiretinas (inferior) | NA | 0.350 | |
| Superior outer sector | 72.9±14.2 (n=28) | 76.9±11.2 (n=32) | 0.24 |
| Superior inner sector | 96.4±16.6 (n=28) | 100.9±11.8 (n=32) | 0.22 |
| Inferior outer sector | 69.6±11.7 (n=28) | 65.8±11.1 (n=32) | 0.20 |
| Inferior inner sector | 88.7±15.8 (n=28) | 88.7±14.0 (n=32) | 0.99 |

DH = disc hemorrhage, NA = not applicable

*Mixed-effects models accounting for the correlation between two hemiretinas within the same eye. Other p-values were by Student's t-test.

§P-values significant using the Benjamini-Hochberg method for controlling the false discovery rate at 5%.

**Table S2.** Baseline total deviation (dB)

| | no DH (n=28) | DH (n=32) | p-value, between groups |
|---|---|---|---|
| Both hemifields combined | -3.5±5.3 (n=56) | -3.8±5.3 (n=64) | 0.82 |
| superior hemifield | -3.2±5.4 (n=28) | -4.3±6.2 (n=32) | 0.48 |
| inferior hemifield | -3.8±5.2 (n=28) | -3.3±4.4 (n=32) | 0.65 |
| p-value, between hemifields | 0.625* | 0.435* | |
| DH+ hemifield | NA | -3.9±5.1 (n=35) | NA |
| DH- hemifield | -3.5±5.3 (n=56) | -3.6±5.7 (n=29) | 0.94* |
| p-value, between hemifields | NA | 0.838* | |
| DH+ superior hemifield | NA | -3.5±5.9 (n=22) | NA |
| DH+ inferior hemifield | NA | -4.6±3.7 (n=13) | NA |
| DH- superior hemifield | -3.2±5.4 (n=28) | -6.1±6.7 (n=10) | 0.19 |
| DH- inferior hemifield | -3.8±5.2 (n=28) | -2.3±4.7 (n=19) | 0.32 |
| p-value, between superior and inferior hemifields (DH+) | NA | 0.518* | |
| p-value, between superior and inferior hemifields (DH-) | 0.625 | 0.068 | |
| p-value, between DH+ and DH- hemifields (superior) | NA | 0.250 | |
| p-value, between DH+ and DH- hemifields (inferior) | NA | 0.131 | |

DH = disc hemorrhage, NA = not applicable

*Mixed-effects models accounting for the correlation between two hemifields within the same eye. Other p-values were by Student's t-test.

None of the p-values were significant using the Benjamini-Hochberg method for controlling the false discovery rate at 5%.
